# Trends in anti-HER2 drugs consumption and influencing factors

**DOI:** 10.3389/fpubh.2022.944071

**Published:** 2022-09-08

**Authors:** Jie Liu, Xiaolei Zhang, Biao Wang, Huizhen Dai, Dahai Dou, Wentong Fang

**Affiliations:** ^1^Department of Pharmacy, Nanjing Pukou Central Hospital, Pukou Branch of Jiangsu Province Hospital, Nanjing, China; ^2^Department of Urology, The First Affiliated Hospital of Nanjing Medical University, Nanjing, China; ^3^Department of Pharmacy, The First Affiliated Hospital of Nanjing Medical University, Nanjing, China; ^4^Department of Pharmacy, Jiangsu Medicine Information Institute, Nanjing, China

**Keywords:** anti-HER2 drug, consumption, national health insurance coverage, national drug price negotiation, generic drug replacement

## Abstract

**Background:**

Human epidermal growth factor receptor 2 (HER2) inhibitors have been approved to treat various cancers with HER2 amplification. The Chinese government has made great efforts to improve the availability and affordability of these drugs. This study aimed to analyze the trends in anti-HER2 drug consumptions in Nanjing from 2012 to 2021, and explore influencing factors.

**Methods:**

Data about use of anti-HER2 drugs in 2012–2021 were extracted from Jiangsu Medicine Information Institute. Six types of anti-HER2 drugs were included. Drug consumption was expressed as defined daily doses (DDDs) and expenditure. Time series analysis was adopted to find trends in consumption, while interrupted time series was used in analyzing the impact of policy on consumption. The correlation between DDDs and defined daily cost (DDC) was analyzed by Pearson's correlation test.

**Results:**

The DDC, DDDs, and expenditure of anti-HER2 drugs changed little from 2012 to 2016. The DDC decreased intermittently, while the DDDs and expenditure of these drugs grew continuously from 2017 to 2021. The anti-HER2 monoclonal antibodies contributed to the majority of total consumption in 2012–2019. The DDDs of anti-HER2 tyrosine kinase inhibitors surpassed the DDDs of monoclonal antibodies in 2020–2021. Trastuzumab was the predominantly prescribed drug in 2012–2019, but the DDDs of pyrotinib surpassed the DDDs of trastuzumab in 2020–2021. The ln value of DDC or self-paid DDC of trastuzumab was negatively correlated with the ln value of its DDDs. The national health insurance coverage (NHIC) and national drug price negotiation policy about anti-HER2 drugs were initiated in 2017. Low-price generics and biosimilar of trastuzumab came into the market in 2020 and 2021, separately. Interrupted time series analysis showed that the DDDs increased significantly after the implementation of NHIC, price negotiation or generic drug replacement.

**Conclusion:**

The consumption of anti-HER2 drugs has significantly increased and their DDC has decreased after the implementation of NHIC, price negotiation, or low-price generic drug replacement since 2017. Further efforts are needed to translate the high consumption into clinical benefits.

## Background

Human epidermal growth factor receptor 2 (HER2) is a member of the epidermal growth factor receptor (EGFR) family. HER2 can form into heterodimers with other members, such as HER1, HER3 and HER4, and acts in the pathogenesis and progression of several human cancers (1, 2). Anti-HER2 drug, as a breakthrough invention, have increased the survival of cancer patients with HER2 amplification (3). Trastuzumab (Herceptin) is the first humanized monoclonal antibody targeting HER2, and has been approved for the treatment of HER2-positive breast cancer and gastric cancer (4). Subsequently, other anti-HER2 drugs, such as pertuzumab, lapatinib, and pyrotinib, have been commercialized to treat cancers with HER2 amplification.

However, the high cost of these drugs limits their access to eligible patients (5). In a survey conducted on breast cancer in Africa, trastuzumab could be provided by 10 out of 19 facilities, but afforded by only 5% of the patients (6). In Jiangsu, a developed province in China, only 33.39% of patients with early-stage breast cancer received trastuzumab in 2010–2013 (7).

The Chinese government has made great efforts to increase the availability and affordability of anti-HER2 drugs. In 2017, trastuzumab and lapatinib were covered by national health insurance with a reimbursement rate of 70–80% (8). Then, low-price generic and biosimilar drugs were introduced into the market, which have potentially increased the accessibility and affordability of anti-HER2 drugs (9). Meanwhile, several rounds of national price negotiations of anticancer drugs have been accomplished, after which the price of trastuzumab and lapatinib were significantly decreased (10). In this light, the consumption of anti-HER2 drugs may have demonstrated new trends in China. The aim of this study was to analyze the consumption trend of anti-HER2 drugs from 2012 to 2021 in Nanjing, the capital city of Jiangsu province and evaluate influencing factors.

## Methods

### Data sources

The data about anti-HER2 drugs consumption were provided by Jiangsu Medicine Information Institute (11, 12). In China, anti-HER2 drugs can only be prescribed by physicians and borrowed from hospital pharmacies by 2021. If these drugs were covered by medical insurance, only drugs sold by hospital pharmacies can be reimbursed before 2022. Hence, the sales in hospital pharmacies could present the consumption by patients. There are 106 hospitals (including secondary and tertiary hospitals) in Nanjing. Each hospital has a designated reporter, usually a pharmacist, who is responsible for registering the consumption of drugs. The designated reporter reports data to the Jiangsu Medicine Information Institute monthly. The reported information for each drug includes dosage form, package dose, manufacturer, price, monthly expenditure, and monthly consumption (in terms of grams). By analysis these data, we found 36 hospitals (33.96%) consumed anti-HER2 drugs in the past 10 years. Hence, these 31 tertiary hospitals and five secondary hospitals were included in our study.

Six kinds of anti-HER2 drugs were used in Nanjing by 2021, including trastuzumab, pertuzumab, inetetamab, trastuzumab-emtansine, lapatinib, and pyrotinib. Trastuzumab is sold as either original (Herceptin) or generic drugs (Zercepac). Inetetamab is a biosimilar drug of trastuzumab. The information of anti-HER2 drugs used in Nanjing is listed in Table 1.

**Table 1:** Information about the anti-HER2 drugs.

**Drugs**	**Kinds**	**Manufacturer**	**Launch date**	**Reimbursement date**	**Price negotiation**
Trastuzumab (original)	mAb	Roche (Switerland)	Sep-98	Jul-17	¥21999.42 to ¥7600.00 (Jul 17)
					¥7600.00 to ¥ 7270.20 (Aug 19)
					¥7270.20 to ¥ 5500.00
Trastuzumab (generic)	mAb	Henlius (China)	Aug-20	Dec-20	¥1688.00
Pertuzumab	mAb	Roche (Switerland)	Jun-12	Nov-19	¥4955.00
Inetetamab	mAb	Sunshine Guojian (China)	Jun-20	Dec-20	¥590.00
Trastuzumab-emtansine	ADC	AstraZeneca (UK)	May-19	_	¥19282.00
Lapatinib	TKI	GSK (UK)	Mar-07	Jul-17	¥121.43 to ¥ 70.00 (Jul 17)
					¥ 70.00 to ¥ 66.70 (Aug 19)
Pyrotinib	TKI		Aug-18	Nov-19	¥ 86.00

### Data analysis

The monthly sales data of anti-HER2 drugs were analyzed. Two analysts (Liu and Dou) were trained to screen and extract the data using a form, including price, dosage, selling time, specifications, pharmaceutical manufacturer. The quality of the data was checked by a supervisor (Fang).

### Utilization analysis of anti-HER2 drugs

Consumption of anti-HER2 drugs was expressed as defined daily doses (DDDs) and expenditure (12, 13). The defined daily dose (DDD) is a statistical unit defined by the WHO Collaborating Centre for Drug Statistics Methodology (14). As there was no standard DDD for anti-cancer medicines, we obtained the data about DDD based on the daily doses and indications from the authoritative specification database. The greater the DDDs, the greater frequency of using the medicine. The expenditure was recorded in Yuan (¥). In our study, DDDs and expenditure were calculated with the following formula:


$$\begin{array}{l} \text{ DDDs = }(\sum (\text{Total dose used in}\\ \text{ number of grams})\text{/DDD})\\ \text{Expenditure = }(\text{retail price per package})\\ \;*(\text{consumption amount in number of package}) \end{array}$$

### Calculation of DDC

Price was expressed as the median defined daily cost (DDC) (15). DDC was the cost of per DDD drug. A higher DDC indicated that the drug was more expensive. The DDC was recorded in Yuan (¥). In our study, DDC were calculated with the following formula:


$$\begin{array}{l} \text{DDC}=\text{expenditure}/\left(\text{the number of DDDs}\right) \end{array}$$

### Analysis of DDDs changes

Interrupted time series (ITS) regression analysis was used to analyze the changes in the DDDs of anti-HER2 drugs in 2012–2021. When it was difficult or impossible to find a control group, the ITS model was designed in a quasi-experimental manner to analyze the longitudinal effects of the interventions. The ITS model could evaluate whether policy intervention had a transient or long-term impact (16). The national health insurance coverage (NHIC) policy, national price negotiation policy, and generic drug replacement were implemented intermittently. Hence, there were several months before the initiation, as well as after the end of policy intervention. To perform independent tests, the trends in DDD changes were expressed in three parts: (i) the slope before policy implementation, (ii) the level during policy intervention, and (iii) the slope after policy implementation. The following ITS model formula was used:


$$\begin{array}{l} {\text{Y}}_{\text{t}}={\text{β}}_{0}+{\text{β}}_{1}\text{T}+{\text{β}}_{2}\text{D}+{\text{β}}_{3}\text{P}+\text{ ε} \end{array}$$

Y_t_ is the monthly consumption measured at each time point (T). T is the time point after the initiation of study (T = 1, 2, 3... 12). D is the dummy variable for the two time periods before and after policy implementation (D = 0 represents the period before policy implementation and D = 1 represents the period after policy implementation). P is the time point after policy intervention (P = 0 indicates before policy intervention and P = 1, 2, 3, 6 indicates after policy intervention). β_0_ is the intercept (which refers to the consumption at the baseline), β_1_ is the slope before the intervention, β _2_ is the level of change during the intervention, β_3_ is the change in the consumption caused by the policy intervention, β_1_ + β_3_ is the slope after the intervention, and ε is the error term (17). The ITS model is presented in Supplementary Figure 1.

The Durbin–Watson test was used to test the first-order autocorrelation of the data (15). It is extremely possible that the observations are independent. The feasible generalized least square method was used to modify the first-order autocorrelation errors if needed (18). Correlation between the lg value of DDDs and the lg value of DDC was analyzed by Pearson's correlation test and linear regression analysis. All analyses were performed using STATA v.14 software (STATA Corporation, College Station, TX, USA), and *p* = 0.05 was considered significant.

## Results

### Trends in anti-HER2 drug consumption

**Figure 1:**
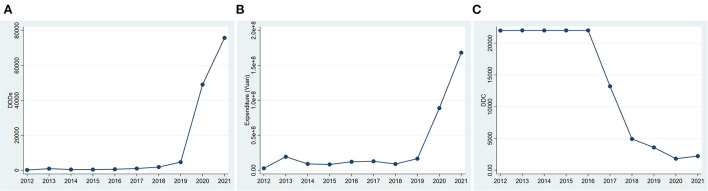
Consumption of anti-HER2 drugs in Nanjing from 2012 to 2021. **(A)** DDDs of anti-HER2 drugs; **(B)** Expenditure of anti-HER2 drugs. **(C)** DDC of anti-HER2 drugs. DDDs, defined daily doses; DDC, defined daily cost.

The DDDs of all anti-HER2 drugs changed slightly from 2012 to 2016, but kept increasing since 2017. The number of DDDs increased by 76.64% in 2017, 86.78% in 2018, 155.31% in 2019, 962.10% in 2020, and 54.46% in 2021, all compared to that in the previous year (Figure 1A). Accordingly, the expenditure also kept increasing significantly from 2017 to 2021 (Figure 1B). The average DDC of all anti-HER2 drugs changed slightly from 2012 to 2016, but decreased markedly from 2017 to 2020 (Figure 1C). The DDC decreased by 39.99% in 2017, 62.71% in 2018, 27.28% in 2019 and 49.41% in 2020, all compared to that in the previous year (Figure 1C). The average DDC changed little in 2021 (Figure 1C).

### Consumption of three generations of anti-HER2 drugs

**Figure 2:**
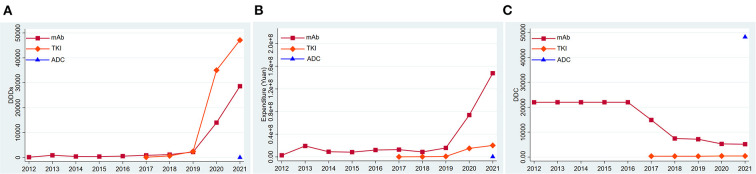
Consumption of three kinds of anti-HER2 drugs in Nanjing from 2012 to 2021. **(A)** DDDs of three kinds of anti-HER2 drugs; **(B)** Expenditure of three kinds of anti-HER2 drugs; **(C)** DDC of three kinds of anti-HER2 drugs. DDDs, defined daily doses; DDC, defined daily cost.

According to their molecular structures, the anti-Her2 drugs fall into three categories: monoclonal antibodies (mAbs), tyrosine kinase inhibitors (TKIs) and antibody-drug conjugates (ADCs). From 2012 to 2016, only anti-HER2 mAbs were used in the market, and its DDDs (Figure 2A), expenditure (Figure 2) and DDC changed little. The DDC of mAbs decreased gradually (Figure 2C), while their number of DDDs increased year by year since 2017 (Figure 2A). Overall, the mAb made up the majority of the total consumption from 2012 to 2018. The TKIs entered the market in 2017. The DDDs of TKIs increased significantly and surpassed the DDDs of mAbs in 2019 (Figure 2A). The expenditure of TKIs had an upward trend (Figure 2B), while its DDC had a downward trend (Figure 2C). An ADC (Trastuzumab-emtansine) was launched in 2021, and its DDC was much higher than those of other anti-HER2 drugs (Figure 2C). The DDDs and expenditure of ADC were much lower than those of mAbs and TKIs (Figures 2A,B).

### Consumption of each type of anti-HER2 drug

**Figure 3:**
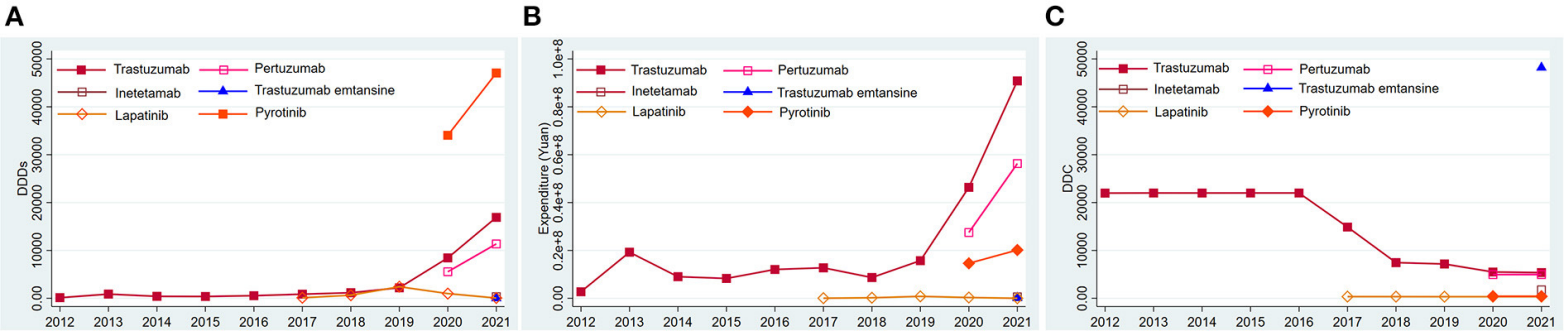
Consumption of six types of anti-HER2 drugs in Nanjing from 2012 to 2021. **(A)** DDDs of six types of anti-HER2 drugs; **(B)** Expenditure of six types of anti-HER2 drugs; **(C)** DDC of six types of anti-HER2 drugs. DDDs, defined daily doses; DDC, defined daily cost.

Trastuzumab was always on the market in the past 10 years. Its DDDs, expenditure, and DDC changed slightly from 2012 to 2016. The DDDs (Figure 3A) and expenditure (Figure 3B) of trastuzumab showed an ascending trend, while its DDC showed a descending trend since 2017 (Figure 3C). Lapatinib came into the market in March 2007, and has been used in Nanjing since 2017. Its DDDs (Figure 3A), expenditure (Figure 3B), and DDC (Figure 3C) changed little from 2017 to 2021 (Figures 3A–C). Pertuzumab and Pyrotinib came into the Nanjing market in 2020, and their DDDs increased by 104.77 and 38.17% separately in 2021 (Figure 3A). Trastuzumab was the predominantly prescribed drug in 2012 to 2019, but the DDDs of Pyrotinib surpassed the DDDs of trastuzumab in 2020 to 2021. Inetetamab and trastuzumab-emtansine came into the market in 2021, but their consumptions were relatively low (Figures 3A,C). The DDC of trastuzumab-emtansine was the highest in all the anti-HER2 drugs (Figure 3C).

### Relationship between DDC and DDDs

**Figure 4:**
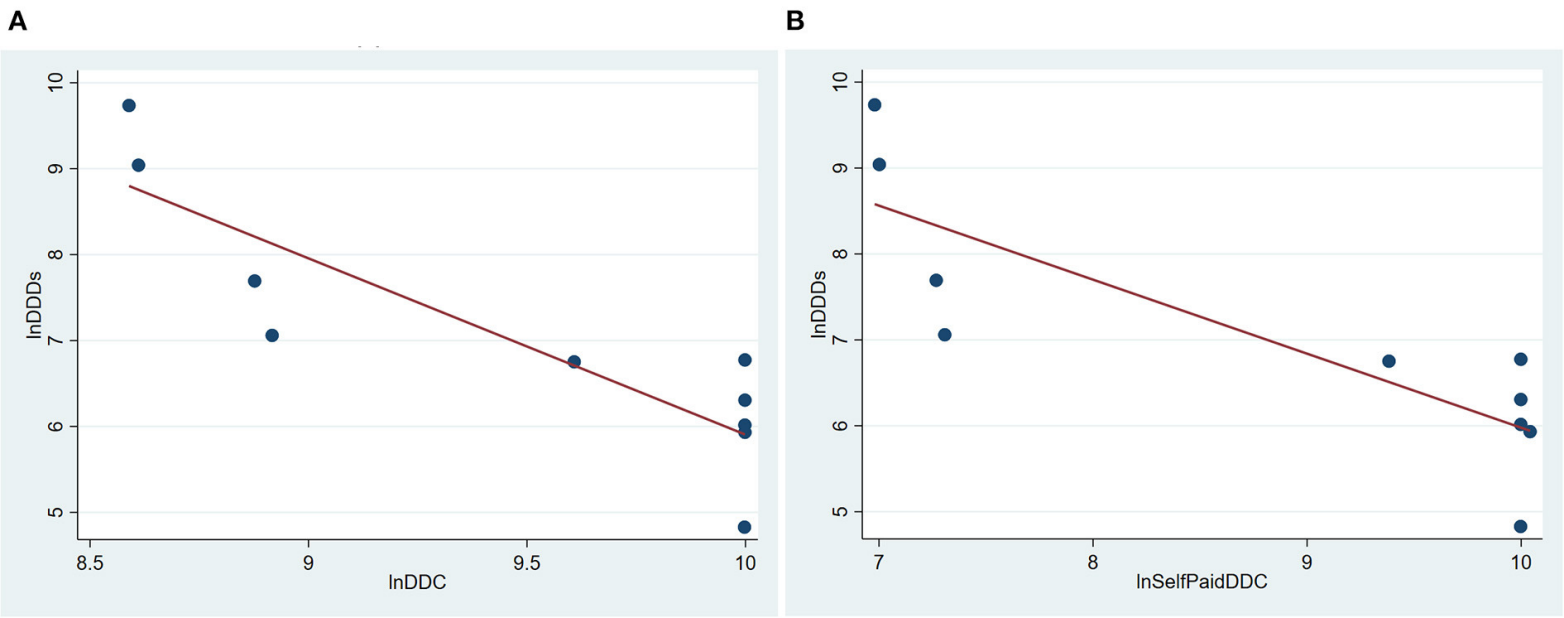
Correlation of DDC and DDDs of trastuzumab in Nanjing from 2012 to 2021. **(A)** Correlation between total DDC and DDDs of trastuzumab in Nanjing from 2012 to 2021; **(B)** Correlation between self-paid DDC and DDDs of trastuzumab in Nanjing from 2012 to 2021. DDDs, defined daily doses; DDC, defined daily cost.

From 2012 to 2021, the price of trastuzumab has been reduced for several times. Hence, we analyzed the relationship between their DDC and DDDs. Its DDC decreased gradually, while DDDs increased continuously since 2017. The ln value of its DDC had a negative correlation with its ln value of DDDs (*R*^2^ = 0.7720, *P* = 0.001) (Figure 4A). As trastuzumab has been enrolled into the national insurance in 2017, self-paid cost (out of pocket cost) was the real expenditure patients paid. A negative correlation existed between the ln value of self-paid DDC and the ln value of DDDs of trastuzumab (*R*^2^ = 0.7119, *P* = 0.002, Figure 4B).

### Factors associated with DDD changes

Previous studies have reported that some policies, such as NHIC, price negotiation, and low-price generics replacement are impactors of drug consumption. As shown in Table 2, the mean DDDs of the anti-HER2 drugs improved after the implementation of NHIC, price negotiation, or low-price generics replacement. Hence, we analyzed their influence on the DDDs by ITS analysis.

**Table 2:** The influence of insurance and price on drug consumption.

**Drug**	**Change**	**Time**	**Average DDDs of 6 months before change (DDDs per month)**	**Average DDDs of 6 months after change (DDDs per month)**	**DDDs change (%)**
Trastuzumab (original drug)	Covered by medical insurance, DDC decreased from ¥21999.42 to ¥7600.00	Jul 2017	59.83	107.00	78.84
Trastuzumab (original drug)	DDC decreased from ¥7600.00 to ¥ 7270.20	Aug 2019	88.33	118.50	34.16
Trastuzumab (original drug)	DDC decreased from ¥7270.20 to ¥ 5500.00	Jan 2020	209.50	387.67	85.05
Trastuzumab (generic drug)	Covered by medical insurance	Dec 2020	-	182.95	-
Pertuzumab	Covered by medical insurance	Nov 2019	-	164.50	-
Inetetamab (biosimilar)	Covered by medical insurance	Dec 2020	-	36.00	-
Trastuzumab-emtansine	-	May 2019	-	-	-
Lapatinib	Covered by medical insurance, DDC decreased from ¥121.43 to ¥ 70.00	Jul 2017	-	49	-
Lapatinib	DDC decreased from ¥ 70.00 to ¥ 66.70	Aug 2019	72.33	303.33	319.37
Pyrotinib	Covered by medical insurance	Nov 2019	-	-	-

**Figure 5:**
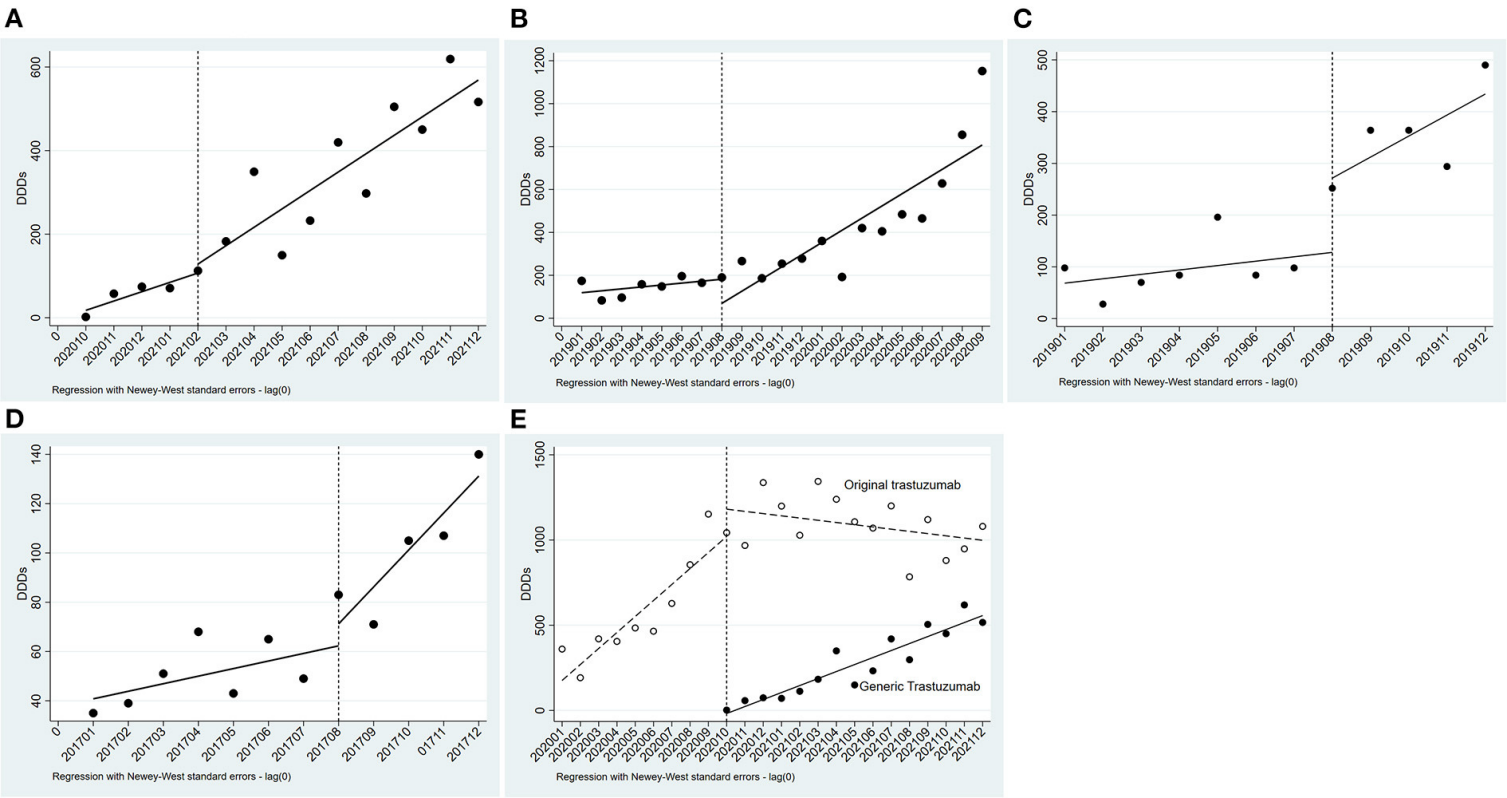
Results of the regression analysis of DDDs before and after policy implementation. **(A)** Regression analysis of DDDs of generic trastuzumab before and after insurance coverage; **(B)** Regression analysis of DDDs of original trastuzumab before and after insurance coverage and price negotiation; **(C)** Regression analysis of DDDs of original trastuzumab before and after price negotiation; **(D)** Regression analysis of DDDs of Lapatinib before and after price negotiation; **(E)** Regression analysis of DDDs of trastuzumab before and after low-price generic replacement.

As show in Table 2, pertuzumab, inetetamab, lapatinib, and pyrotinib were unavailable before the initiation of NHIC; original trastuzumab was subjected to NHIC and price negotiation synchronously in July 2017; trastuzumab-emtansine remained out of covered NHIC by 2021. Hence, only the effect of NIHC on the DDDs of generic trastuzumab (Zercepac) was analyzed by ITS analysis. Zercepac came into the Nanjing market in October 2020 and included by the NHIC in December 2020, after which its DDDs increased significantly (*P* < 0.001, Figure 5A).

The prices of original trastuzumab (Herceptin) has been negotiated for rounds. In July 2017, Herceptin had a great price drop and was covered by health insurance, with a reimbursement rate of 70%. The time series was divided into two parts. As indicated by the results in Table 2, the DDDs of trastuzumab (Herceptin) increased after July 2017. After the initiation of NHIC and price negotiation policy, its DDDs significantly increased (*P* = 0.021, Figure 5B). This was the synergetic effect of NHIC and price negotiation. In August 2019, Herceptin underwent the second round of price negotiation, and its DDC had a slight decrease (from ¥7600.00 to ¥ 7270.20). Hereafer, its DDDs increased, but did not reach statistical difference (*P* = 0.285, Figure 5C). A similar trend was found in the DDDs of lapatinib (*P* = 0.319, Figure 5D) when its DDC decreased from ¥ 70.00 to ¥ 66.70 in Aug2019.

Low-price generic drug replacement showed a significant effect on the consumption of trastuzumab. Generic trastuzumab (Zercepac) came into the Nanjing market in October 2020. Thereafter, the number of monthly DDDs of generic trastuzumab increased significantly, and reached 516.5 in December 2021, which was about half of that of the original drug. Meanwhile, the DDDs of original trastuzumab (Herceptin) had a decreasing trend (*P* < 0.001, Figure 5E).

## Discussion

Our study showed the obvious trends in the consumption of anti-HER2 drugs in Nanjing from 2012 to 2021. Reimbursement, price negotiation, and generic drug replacement all increased their consumption. Our findings provide valuable evidence for the government and health institutes to adopt measures to improve drug availability and affordability.

Trastuzumab (Herceptin) is the first approved drug targeting HER2, and the only used in Nanjing before 2017. Previous studies have proved that its high price limits the patients' access to trastuzumab in underdeveloped areas without reimbursement policy (5–7). Lammers et al. (5) identified potential barriers to the expansion of trastuzumab use in the United States, Mexico, Turkey, Russia and Brazil via physician-oriented survey. Out of insurance coverage, no commercialized drug, and high cost were main barriers restricting the consumption of trastuzumab. In our study, trastuzumab was not covered by the national health insurance system until July 2017. From 2012 to 2016, the average cost of trastuzumab was ¥21999.42 per cycle (21 days), which was far beyond the average household income in Nanjing during the same period (19). This may explain the low and unchanged consumption of trastuzumab in 2012–2016.

DDC has been used as an efficient indicator in nearly one third of studies about drug consumption in China (13, 15). These studies have provided valuable advice for price policy-making of pharmaceutical products on the market (13). Our previous study showed that the DDC of EGFR tyrosine kinase inhibitors significantly affect their consumption (12). Hence, the relation between DDC and DDDs was analyzed in this study. As expected, the ln value of DDC was negatively correlated with the ln value of DDDs (Figure 4). Fortunately, efforts have been taken to reduce drug cost, such as reimbursement policy, national price negotiation, generic drug replacement, low-price drug replacement.

The effect of reimbursement policy on drug consumption has been extensively researched. Policies, such as MGEN plan in French (20), pharmacare programs in Canada (21), national health insurance in Japan (22), Medicare Part D (23) and Medicaid in America (24), have increased drug consumption and decreased out-of-pocket costs. China built up its basic health insurance system in 2009, which expanded the coverage and increased drug availability. In 2017, the system was further enhanced by the price negotiation and mandatory reimbursement policies. In our study, original trastuzumab (Herceptin) and generic trastuzumab (Zercepac) ran into the NHIC in July 2017 (8) and December 2020 (10), separately. The NHIC significantly increased the consumption of trastuzumab (Figures 5A,B and Table 2).

In response to increases in drug prices during the past few decades, many countries have implemented policies of price negotiation. These polices have significantly reduce drug price and increased drug consumption in Italy, France (25), America (26), and Germany (27). The Chinese government has implemented this policy in 2017, the DDC of the 15 targeted anticancer drugs dropped from US$169.24 to US$71.21 (28). Price negotiations have reduce DDC and increased the DDDs of anti-HER2 drugs in China (Figures 5B–D and Table 2).

Low-price generic or biosimilar drug replacement can reduce the cost and increase the consumption. A study has been conducted to compare the costs of biosimilars and innovator biologics (five cycles in total) in India, estimating that the use of biosimilars would save about 843 million U.S. dollars yearly (29). Likewise, introducing generics and biosimilars may overcome the barriers limiting the use of trastuzumab. In our study, available generic trastuzumab (Zercepac) significantly decreased the DDC and increased the total consumption of trastuzumab in Nanjing (Figure 5E and Table 2).

There are some limitations in our study. First, the term “consumption” meant the quantity of drugs prescribed, but not drugs administered. Second, the prevalence of HER2-positive cancer was not available, so the association of increased consumption with cancer prevalence needs further analysis. Third, we did not analyze the prescription switch between anti-HER2 drugs after reimbursement and price negotiation. Fourth, we studied the consumption trend of the drugs without treatment efficacy.

## Conclusion

The consumption of anti-HER2 drugs has increased significantly since 2017 in Nanjing, mainly due to the implementation of NHIC, price negotiation, or low-price generic drug replacement. Further efforts are needed to translate the higher consumption of anti-HER2 drugs into clinical benefits.

## Data availability statement

The original contributions presented in the study are included in the article/Supplementary material, further inquiries can be directed to the corresponding authors.

## Author contributions

JL contributed to the initial drafting of the manuscript. HD and DD extract the data. XZ and BW made the ITS analysis. WF design this study and revised the manuscript. All authors contributed to the article and approved the submitted version.

## Conflict of interest

The authors declare that the research was conducted in the absence of any commercial or financial relationships that could be construed as a potential conflict of interest.

## Publisher's note

All claims expressed in this article are solely those of the authors and do not necessarily represent those of their affiliated organizations, or those of the publisher, the editors and the reviewers. Any product that may be evaluated in this article, or claim that may be made by its manufacturer, is not guaranteed or endorsed by the publisher.
