# Supplementary figures and images for: Trends in anti-HER2 drugs consumption and influencing factors
Source: Front Public Health. 2022 Sep 8;10:944071. doi: 10.3389/fpubh.2022.944071 (PMC9493110; doi:10.3389/fpubh.2022.944071)

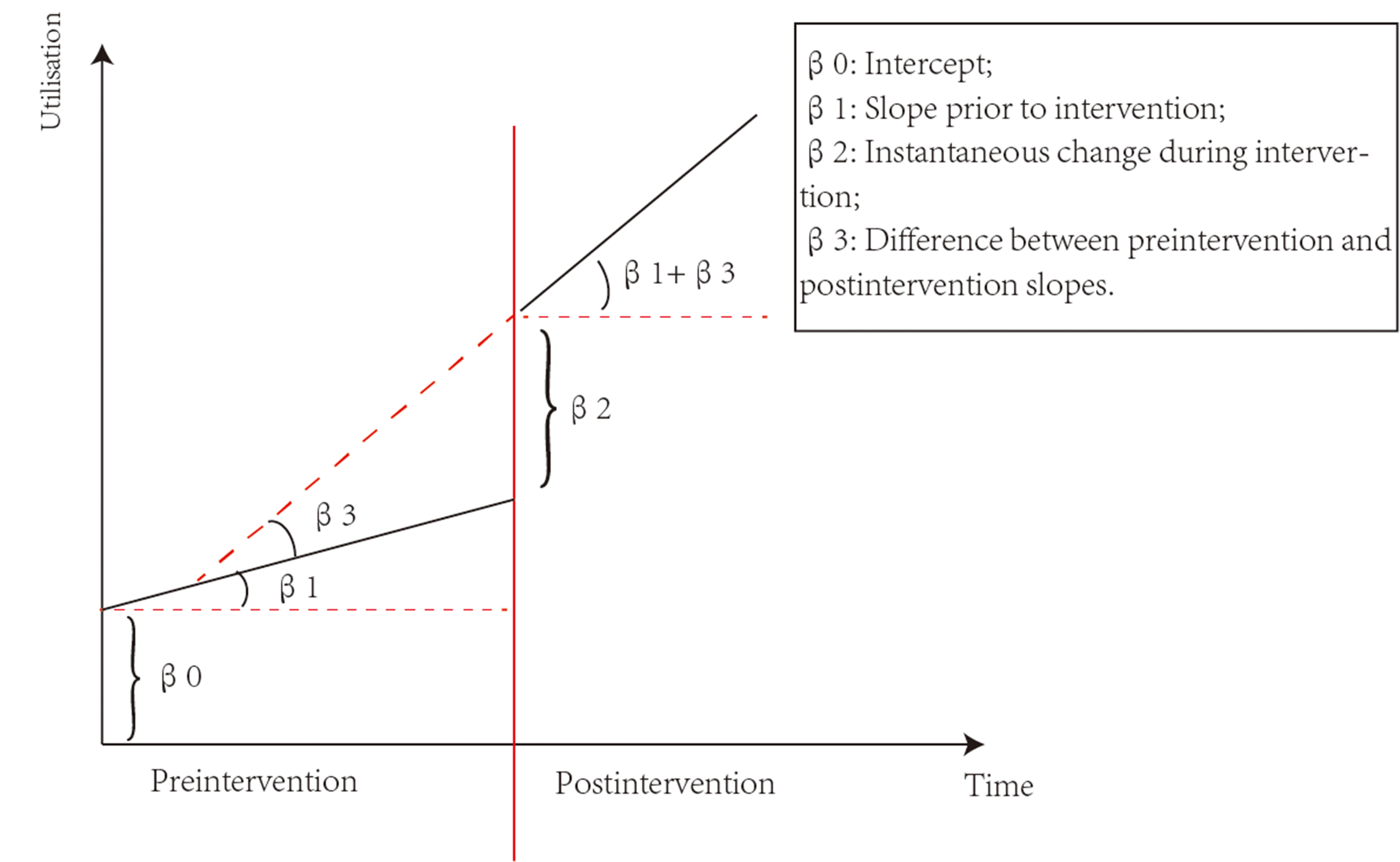

Supplement: Supplementary file 1 [file Image_1.TIF]
